# Thiamine deficiency disorders: a clinical perspective

**DOI:** 10.1111/nyas.14536

**Published:** 2020-12-10

**Authors:** Taryn J. Smith, Casey R. Johnson, Roshine Koshy, Sonja Y. Hess, Umar A. Qureshi, Mimi Lhamu Mynak, Philip R. Fischer

**Affiliations:** ^1^ Institute for Global Nutrition, University of California Davis Davis California; ^2^ Pediatric and Adolescent Medicine Mayo Clinic Rochester Minnesota; ^3^ Makunda Christian Leprosy and General Hospital Karimganj Assam India; ^4^ G.B. Pant Hospital Government Medical College, Srinagar Jammu and Kashmir India; ^5^ Jigme Dorji Wangchuck National Referral Hospital Thimphu Bhutan

**Keywords:** vitamin B_1_, beriberi, malnutrition, cardiomyopathy, encephalopathy

## Abstract

Thiamine is an essential water‐soluble vitamin that plays an important role in energy metabolism. Thiamine deficiency presents many challenges to clinicians, in part due to the broad clinical spectrum, referred to as thiamine deficiency disorders (TDDs), affecting the metabolic, neurologic, cardiovascular, respiratory, gastrointestinal, and musculoskeletal systems. Concurrent illnesses and overlapping signs and symptoms with other disorders can further complicate this. As such, TDDs are frequently misdiagnosed and treatment opportunities missed, with fatal consequences or permanent neurologic sequelae. In the absence of specific diagnostic tests, a low threshold of clinical suspicion and early therapeutic thiamine is currently the best approach. Even in severe cases, rapid clinical improvement can occur within hours or days, with neurological involvement possibly requiring higher doses and a longer recovery time. Active research aims to help better identify patients with thiamine‐responsive disorders and future research is needed to determine effective dosing regimens for the various clinical presentations of TDDs. Understanding the clinical diagnosis and global burden of thiamine deficiency will help to implement national surveillance and population‐level prevention programs, with education to sensitize clinicians to TDDs. With concerted effort, the morbidity and mortality related to thiamine deficiency can be reduced.

## Historical perspective

Beriberi is the popular name for a group of disorders due to thiamine deficiency. According to (unconfirmed) legend, the name “beriberi” was commonly used because when afflicted patients in Sri Lanka who were suffering from neurologic disability were asked to move, they responded in the Sinhalese language with “beriberi,” which translates to “I cannot. I cannot.” Wherever the initial patients were seen and, however, they were described, infants with thiamine deficiency were subsequently noted to have life‐threatening heart failure, often with wet lungs and peripheral edema. With this, the term *beriberi* was subdivided into two forms, with “wet beriberi” referring to infantile thiamine deficiency (wet referring to the fluid in the lungs and swollen extremities) and “dry beriberi” referring to older children and adults with neurologic problems.

As time went on, individuals in various settings with specific circumstances were also found to be suffering from thiamine deficiency. The adult form of dry beriberi was thought to include both sensory and motor forms of peripheral neuropathy as well as a central nervous system (CNS) form with encephalopathy (altered or reduced cognition and alertness). Occurring mostly in alcohol‐abusing adults, the encephalopathic form of beriberi is called Wernicke–Korsakoff syndrome.^1^ Heart failure seemed to be the predominant problem in thiamine‐deficient infants, but some also lost their voices and were said to have “aphonic beriberi.”^2^ Infants (and, rarely, adults) with severe lactic acidosis and cardiac collapse were said to have “Shoshin beriberi” (from the Japanese words “sho,” meaning acute damage, and “shin,” meaning heart).^3^

As further subdivisions of the various forms of beriberi were recognized, the dividing lines between the various forms also became blurred. Some infants had encephalopathy, and some adults had heart failure. Some infants had encephalitic presentations (with signs of brain inflammation) along with altered CNS function. In Africa, there have been clusters of people with beriberi‐related ataxia (impaired balance and coordination), and others with spasticity (increased muscle tightness) with paralysis.^4^, ^5^, ^6^ More recently, infants who were accidentally deprived of thiamine because of a formula manufacturing error in Israel had developmental delay and, in some cases, hearing loss and death.^7^, ^8^

To further increase the complexity of the clinical situation, thiamine deficiency may present in association with an infection, with some of the presenting features of the illness being due to combined effects of the infection and the thiamine deficiency. Also, in a group of symptomatically similar infants presenting with what clinically seemed to be classic wet beriberi with heart failure, only a small minority had echocardiographic and blood test (B‐type natriuretic peptide) abnormalities.^9^ It can be assumed that other factors might contribute to, alter, or even mask the diagnostic features of the thiamine deficiency. While thiamine deficiency is clinically diagnosed as an isolated micronutrient deficiency, it could certainly coexist with presentation‐altering deficiencies of other vitamins and minerals (e.g., magnesium) or even with generalized severe acute malnutrition (SAM).

Thus, as the clinical manifestations of thiamine deficiency have been better elucidated, it has become increasingly difficult to neatly categorize the various patterns by which thiamine deficiency presents clinically. This led to the term *thiamine deficiency disorders* (TDDs) in an effort to better describe the spectrum of overlapping clinical presentations attributable to thiamine deficiency during the life course.^10^

In resource‐limited settings, thiamine deficiency is usually seen in older children and adults with thiamine‐restricted diets, or in infants of thiamine‐deprived breastfeeding mothers. Practically, this occurs when diets are monotonous and the main staple consists of polished white rice (with thiamine in the husk of the rice being removed during the polishing or preparation process) or cassava. In resource‐rich countries with fortified foods and diverse diets, thiamine deficiency is more often seen in individuals with medical comorbidities, such as alcoholism, diets restricted to heavily processed foods, renal disease, eating disorders, bariatric surgery, and dependence on parenteral nutrition.^11^ Thiamine deficiency has also been reported in intensive care unit settings when patients are critically ill with infections and other illness.^11^

For clinicians, it is particularly challenging to deal with TDDs for several reasons. First, the symptoms and signs related to thiamine deficiency are often not specific and can overlap with symptoms and signs of unrelated disorders. Second, patients can present with concurrent thiamine deficiency and a different condition, with alterations in the clinical presentation. Third, testing for thiamine deficiency is not readily available in areas where thiamine deficiency is prevalent, and awaiting laboratory results in any setting can lead to delays in diagnosis, with potentially fatal or irreversible consequences. Furthermore, neither the best biomarker nor the normal range cutoffs of biomarker tests have been definitively established. Fourth, treatment regimens have not been fully elucidated. Finally, there are national and policy differences in how clinicians in various areas can deal with thiamine deficiency.

Based on this brief historical context, we review the pathophysiology, clinical manifestations, diagnosis, treatment, and current global situation in regard to TDDs and propose some future directions. This clinical perspective is intended to complement other recent biology‐based^10^ and public health–centered^12^ reviews.

## Pathophysiology and clinical manifestations

Here, we discuss the pathophysiologic mechanisms by which deficiency of thiamine affects various organ systems and describe the various presenting symptoms and signs that can present individually or in combination in various population subgroups afflicted by thiamine deficiency.

### Metabolic

Thiamine forms an essential cofactor in the metabolism of carbohydrates and amino acids. Thiamine diphosphate (ThDP), the active metabolite, is a cofactor in the pyruvate dehydrogenase complex, the α‐ketoglutarate dehydrogenase complex, the branched chain α‐keto acid dehydrogenase complex, the pentose phosphate pathway (cytosolic transketolase), and in α‐oxidation of phytanic acid (2‐hydroxyacyl‐CoA lyase).^13^ In a thiamine‐deficient state, these enzymes limit supply to and cycling of the Krebs cycle, resulting in decreased adenosine triphosphate (ATP) synthesis, oxidative damage, and cell death.^13^ Metabolic disturbances in thiamine deficiency lead to a metabolic acidosis, and laboratory evaluation will often reveal an elevated lactate concentration.^14^ Thiamine is primarily transported in erythrocytes and delivered to areas of high metabolic demand in the brain, heart, liver, pancreas, muscles, and nerves. Accordingly, these systems are the first to be affected by thiamine deficiency and are discussed below. It is also worth noting that if the metabolic disturbances of thiamine deficiency persist, the chronic energy deficit leads to significant malnutrition and weight loss.

### Neurologic

Neurologic toxicity in a thiamine‐deficient state is multifactorial. Metabolic derangements disrupt the energy supply to neurons and increase oxidative stress. The resultant oxidative state and lactic acid buildup provoke abnormalities in the blood–brain barrier.^15^ Several functional changes in neurotransmission occur in thiamine deficiency, most notably among the glutamatergic and GABAergic systems, resulting in a toxic neuroexcitatory state.^16^, ^17^, ^18^ Derangements in the pentose phosphate pathway lead to decreased neuronal myelination and subsequent signaling impairments.^19^

Early signs of thiamine deficiency include peripheral neuropathies in adults and adolescents and fussiness and irritability in infants. Weakness, nystagmus, ophthalmoplegia, ataxia, and cognitive impairment accompany progression of the disease. Infants may be noted to have a lack of tone. Neurologic symptoms are often reversed quickly with treatment, but lasting effects may be seen in severe cases or with delayed treatment. Thiamine levels are notably low in some patients with acute stroke, and it is not known just how thiamine deficiency relates to the occurrence of stroke.^20^

Brain magnetic resonance imaging (MRI) of severely thiamine‐depleted infants may reveal lesions in areas of particularly high metabolic demand, notably the periaqueductal area, basal ganglia, thalami, mammillary bodies, brain stem, and the frontal region. Magnetic resonance spectroscopy may reveal a lactate doublet in active lesions.^21^ Head ultrasound scans of infants with encephalopathy have demonstrated hyperechoic basal ganglia.^22^ Interestingly, the brain changes with beriberi are very similar to the changes seen in individuals with genetic alterations in thiamine function due to mutations in specific genes (including *SLC19A3*, *SLC25A19*, and *TPK1*).^23^ Long‐term follow‐up may reveal atrophy or parenchymal loss of affected regions.^21^ Electromyography in thiamine‐deficient adults with peripheral neuropathy is consistent with axonal neuropathy, and peripheral nerve biopsy reveals nerve degeneration.^24^

### Cardiovascular

Cardiomyocytes require a constant supply of energy, and aberrations in aerobic respiration pathways caused by thiamine deficiency interrupt normal heart functioning. This is compounded by endovascular dysfunction as a result of thiamine‐dependent nitric oxide synthase,^25^ an enzyme which is also implicated in the lungs of patients with pulmonary hypertension.^26^ So, too, in thiamine deficiency, the culmination of the disturbances is pulmonary hypertension and often right‐predominant heart failure.^27^, ^28^ Importantly, heart failure may be low or high output in TDDs. Adults with cardiac involvement may have lower extremity edema and congestion of the liver and spleen; edema in infants is often more generalized, but also includes hepatosplenic congestion. Ventricular dilatation and pulmonary hypertension can result in valvular insufficiency, especially tricuspic regurgitation. Cardiomyocyte dysfunction with thiamine deficiency theoretically increases the risk of cardiac arrhythmias; however, this has only been documented experimentally in thiamine‐deficient rats.^29^ In infants, tachycardia has been observed almost universally.^27^

Chest X‐ray may reveal cardiomegaly. Echocardiography of severely deficient infants can show dilated ventricles (especially the right ventricle, often with pulmonary hypertension), a D‐shaped left ventricle, and significantly reduced ejection fraction. Prompt treatment with thiamine will reverse the effects dramatically over a period of hours to days.^9^ Electrocardiography may reveal right‐axis deviation, nonspecific ST and T‐wave abnormalities, or T‐wave inversions.^30^

### Respiratory

TDDs are often associated with tachypnea.^27^, ^28^, ^31^ This may be the result of metabolic acidosis and resultant respiratory compensation. Pulmonary edema in cases of fulminant heart failure will also cause tachypnea. Pulmonary hypertension may exacerbate dyspnea and overall respiratory condition in some cases. In early stage of the illness, infants may present with unexplained hypoxia and irritability. A chest X‐ray may reveal increased pulmonary markings.

### Gastrointestinal

Pancreatic acinar cells are responsive to thiamine deficiency, dramatically decreasing the secretion of digestive enzymes.^32^ Thiamine deficiency also causes depletion of ATP in the liver, which may result in elevated liver enzymes, even in the absence of hepatic congestion secondary to right‐sided heart failure. Although not reported in the literature, the myenteric plexus is likely to be involved in cases of thiamine deficiency, as are other neurological structures. Vomiting, constipation, diarrhea, and anorexia can occur with acute thiamine deficiency–related illness. Vomiting, in particular, has been linked to lower erythrocyte transketolase (ETK) activity.^33^

Adults may experience severe abdominal pain, nausea, vomiting, and anorexia; infants may have anorexia and vomiting. Administration of thiamine quickly reverses these symptoms.^34^

### Musculoskeletal

As thiamine deficiency progresses, extreme loss of muscle mass can be observed. Early reports of the phenomenon particularly indicate severe wasting of the gastrocnemii. Adults demonstrate considerable weakness related to muscle loss and peripheral neuropathy. A squat test is a simple measure of weakness that has been used in low‐ and middle‐income countries (LMICs) to screen for thiamine deficiency.^35^, ^36^, ^37^, ^38^

Table 1 notes some of the key clinical features of TDDs and the patient populations in which they occur. While the framework of the table can be helpful, one must remember that symptoms and signs can be multifactorial. For instance, aphonia can be due to decreased pulmonary air movement and/or to recurrent laryngeal neuropathy.^39^ In Table 1, cardiac and pulmonary features are combined, partly because pulmonary hypertension can be a result of pulmonary vasculature dysfunction and then can subsequently lead to heart failure, or heart failure can lead to pulmonary hypertension. Though not explicitly shown in Table 1, tachypnea can concurrently result from metabolic acidosis and/or pulmonary disease and/or heart failure. Thiamine deficiency can cause multiple interaction pathologies, symptoms, and signs.

**Table 1:** Organ system manifestations of thiamine deficiency as seen at different ages in various geographical areas

Geographic setting	Metabolic	Cardiorespiratory		Neurologic				Musculoskeletal	Gastro‐intestinal
	Acidosis	Heart failure	Dysphonia	Encephalopathy	Developmental delay and hearing loss	Peripheral neuropathy	Ataxia	Weakness and atrophy	Vomiting
Southeast Asia		I^9^, ^43^ A^79^	I^2^, ^79^	I^79^					I^33^
Bhutan		I*^a^*		I^103^		C^104^ A*^a^*		C^104^	I*^a^*
India	I^28^, ^53^	I^28^, ^56^	I^56^	I^22^		A^70^			
Kiribati		I^38^				A^38^		A^38^	
Israel	I^105^				I^7^, ^8^, ^49^				
Africa		I^48^, ^106^ A^107^		C^108^			C^108^ A^4^, ^5^, ^6^		A^107^
Americas		A^109^	A^110^			A^111^		A^111^, ^112^	
Europe	A^113^								
Possible predisposing factors	Genetic;^114^A – alcoholism;^113^ thiamine‐deficient parenteral nutrition formulas^115^	I – breastfed infants of women with thiamine‐insufficient diet;^10^, ^116^ A – ingestion of antithiamine factors^91^, ^117^		A – alcoholism^79^	I – infant formula manufacturing error^105^	A – following bariatric surgery;^111^ pregnancy^70^	C and A – ingestion of the roasted larvae of *Anaphae venata* ^108^, ^118^		A – hyperemesis gravidarum, drug‐induced hyperlactatemia, and disease‐related malnutrition^119^

A, adult; C, child; I, infant.

Note: This table provides representative examples of key information. It should be considered neither restrictive nor exhaustive.

^*a*^ M.L. Mynak, personal communication.

## Diagnosis of TDDs

The diagnosis of thiamine deficiency varies depending on the clinical presentation and the resources available to support the diagnosis. Nonetheless, some explanation of terms can be useful.

Thiamine insufficiency refers to a diet, not to people, that lacks adequate amounts of thiamine to support essential bodily functions. Subject to accurate recall, dietary recall and food frequency histories can be obtained, and calculations using food composition databases can estimate the average daily thiamine intake. Thus, average daily thiamine intake in thiamine‐insufficient diets is well below the values shown in tables of recommended intakes. Of course, the thiamine intake of exclusively breastfed infants depends on the thiamine intake and status of the mother.^40^

Thiamine deficiency refers to a state in individuals who have subnormal amounts (ThDP level) or function (ETK activity) of thiamine, compared with healthy members of the population. Thus, thiamine deficiency is a laboratory‐based diagnosis that is independent of signs and symptoms of disease. There are two main biomarkers of thiamine status: the ThDP level and the ETK activity coefficient.^10^ Typically, ThDP levels are between 70 and 180 nmol/L of whole blood in healthy individuals.^10^ Conversely, ETK activity reflects the function of thiamine within red blood cells. Typically, the ETK activity coefficient is reported, which is the ratio between the ETK activity after adding exogenous ThDP divided by the baseline ETK activity.^10^ Although there is no universal consensus, a ratio of >1.25 is suggestive of thiamine deficiency. Reference ranges of ThDP and other forms of thiamine have typically been determined in generally healthy Western populations.^41^, ^42^ It is not clear how these levels might relate to populations in higher risk settings. The fact that individuals both with and without clinical signs of thiamine deficiency have been shown to have low thiamine status complicates the interpretation of thiamine biomarkers and understanding which biomarker better identifies thiamine deficiency.^33^, ^43^ Although the quantitative degree of deficiency may relate to the presenting findings, as noted in Cambodia when, among infants who seemed to have wet beriberi, only the infants with the very lowest ThDP levels had echocardiographic findings of right heart failure,^9^ it is not clear how and how much the actual thiamine level relates to clinical symptoms. By contrast, basal ETK activity was the only thiamine biomarker predictive of beriberi among hospitalized infants in Laos.^44^ In spite of these caveats, when a thiamine level or functional activity test yields results below a population‐based standard, the individual is said to be thiamine deficient. Ideally, all thiamine‐deficient individuals would be identified and treated so that their deficiency could be corrected before it triggers symptomatic illness or subtle adverse sequelae.

TDDs refer to clinical symptoms that are associated with and are, presumably, due to thiamine deficiency. In the absence of testing for ThDP levels or transketolase activity, a clinician might presume the diagnosis of thiamine deficiency based on the clinical setting. Symptoms suggestive of thiamine deficiency include difficulty breathing, altered mental status, loss of voice, severe fatigue, weakness, abnormal eye movements, altered cognition, abdominal discomfort, vomiting, nausea, and loss of appetite. Signs possibly representing thiamine deficiency on physical examination include tachypnea, tachycardia, crackles on lung auscultation, hepatomegaly, aphonia (or a whispering hoarse voice), altered mental status, weakness, abnormal eye movements, hypertonicity, and ataxia. More chronically, developmental delay in young children and difficulty ambulating in older children and adults could be signs of thiamine deficiency. Helpful test results suggesting possible thiamine deficiency could include increased pulmonary markings on chest X‐rays, pulmonary hypertension and reduced ventricular function on echocardiography, and hyperdense abnormalities of the basal ganglia on cranial ultrasound scan or MRI. Elevated lactate levels serve as a nonspecific indicator of metabolic imbalance but could turn out to be indicative of thiamine deficiency in some patients.^45^

Thiamine‐responsive disorders are clinical conditions that improve with the administration of thiamine. The term implies, but is not proof of, thiamine deficiency. Moreover, this term depends on the patient having symptoms and signs attributed to the thiamine deficiency that do, indeed, resolve with treatment. An ongoing study among infants and young children in Laos is determining how best to use presenting clinical features in deciding whether treatment with thiamine is indicated.^46^ Of course, some thiamine‐deficient individuals, perhaps those with CNS changes without acute encephalopathy, will have permanent, incompletely reversible sequelae of their deficiency and might not respond to the administration of thiamine.

With that background, there are practical challenges. In most parts of the world where thiamine deficiency is known to be common, there is no readily available test to confirm thiamine deficiency. Even when tests are available, there is no consensus on the normal ranges for these biomarkers of thiamine status. Furthermore, the absence of clinically apparent response to thiamine administration does not necessarily imply that the patient was not deficient in thiamine and should not have been treated.

Thus, practically, in the absence of thiamine testing, the diagnosis of either a TDD or a thiamine‐responsive disorder is based on a clinician's interpretation of the constellation of symptoms with which the patient presents, the perceived risk of that patient having an underlying thiamine‐insufficient diet, and the response to initial treatment. A hypothetical (yet commonly seen) example can illustrate some of the challenges in diagnosing thiamine‐related disorders. Imagine that a 2‐month‐old infant presents with rapid breathing, tachycardia, noisy wet‐sounding lungs, hypoxia, and a mildly enlarged (or downwardly displaced) liver. A clinician familiar with presentations of beriberi in Southeast Asia will appropriately see this infant as perfectly typical of what was classically called wet beriberi. At the same time, a clinician familiar with bronchiolitis in North America will see this infant as typical of those with respiratory syncytial virus infection. Another wise clinician who is familiar with both conditions could rightly assume that the patient has both illnesses at the same time. If the infant is exclusively breastfed by a woman living in a rural Southeast Asian village who has a restrictive diet consisting of mostly polished rice and who also reports tingling fingers suggestive of peripheral neuropathy, the likelihood of thiamine deficiency causing the infant's illness increases. If, however, the same infant was breastfed by a nutritionally replete asymptomatic woman in North America, thiamine deficiency would seem to be an improbable cause of the illness. Having access to a blood test for ThDP would clarify whether or not there is actual thiamine deficiency, but it still would not prove whether the symptoms were from thiamine deficiency or from just the viral infection or a combination of the two. The viral illness would resolve with supportive care, and the thiamine deficiency would resolve with administration of thiamine; if the infant receives both supportive care and thiamine, it might be difficult to say whether the infant actually responded clinically to the thiamine part of treatment or not.^47^ Recognizing these challenges and waiting for better clinical determinations of TDDs and widely available thiamine testing, clinicians will still make diagnoses and determine treatment plans by considering the presentation, nutritional background, and clinical course of the patient.

## Treatment and prevention of TDDs

Accurate and timely identification of TDDs is required in order to prevent the severe adverse outcomes associated with thiamine‐deficient states, and thiamine administration rapidly alleviates some of the clinical signs and symptoms of deficiency. However, owing to the nonspecific and highly variable clinical presentations of TDDs and the lack of consensus on clinical case definitions, this poses a significant challenge for clinicians. Therefore, a high degree of clinical suspicion and prompt treatment is recommended in high‐risk populations, especially since thiamine treatment is both safe and inexpensive.^48^ If not promptly treated, TDDs can rapidly lead to death from heart failure and irreversible neurologic implications. If left untreated, acute Wernicke's encephalopathy can progress to chronic Wernicke–Korsakoff syndrome, leading to impaired memory and cognitive functions, and coma and death can occur in severe cases.^13^ Long‐term follow‐up of Israeli children exposed to an infant formula that erroneously omitted thiamine and who demonstrated overt signs of TDDs found that they exhibited delayed language development in early childhood and motor and cognitive sequelae.^8^, ^49^ Furthermore, gross and fine motor delays were also found in children followed up at 5–6 years of age who were exposed to the formula but were asymptomatic.^50^ As neurodevelopmental deficits were seen even in asymptomatic children with subclinical thiamine deficiency, this highlights the need to consider preventive strategies, such as supplementation or food fortification, in at‐risk population groups.

### Treatment in clinical settings

There are currently insufficient data on the optimal mode of administration, dose, and duration of treatment for TDDs among different age groups with differing clinical presentations. Recommendations for various target population groups are summarized in Table 2.

**Table 2:** Recommendations for the treatment of thiamine deficiency

Organization	Target group/condition	Recommendation
WHO (1999)^79^	Mild deficiency states (including lactating women at risk of inadequate intakes)	10 mg daily oral dose for 1 week, followed by 3–5 mg daily oral dose for 6 weeks
	Infantile thiamine deficiency (severe heart failure, convulsions, or coma)	25–50 mg IV immediately, followed by 10 mg IM daily for 10 days, then 3–5 mg daily oral dose for 6 weeks
	Critically ill adults	50–100 mg IV immediately, followed by 3–5 mg daily oral dose for 6 weeks
Royal College of Physicians (2001)^120^	Alcoholism	Inpatient settings: 500 mg IV thiamine, once or twice daily, for 3–5 days, followed by 50 mg oral thiamine four times daily at discharge if there is evidence of cognitive impairment Community settings: 200 mg oral thiamine four times daily, together with a B‐complex tablet containing 30 mg thiamine
European Federation of Neurological Societies (2010)^67^	Wernicke's encephalopathy	200 mg three times daily, preferably IV
WHO Western Pacific Region (2017)^57^	Infants and children with heart failure as a result of thiamine deficiency	25 mg IV and 25 mg IM, then 25 mg daily until the child can eat, followed by 10 mg oral supplementation for 2–3 weeks. Treat the mother at the same time with 100 mg two times per day for 1 month until mother can eat a more diverse diet

IM, intramuscular; IV, intravenous.

#### Infants and children

There are no evidence‐based recommendations for treating TDDs among infants and children, and the doses used in clinical practice are empirically derived and vary widely (50–1500 mg/day), depending on clinical manifestations and the locally available preparations and drug dilutions in low‐resource settings (Table 3). The route of administration is determined by clinical severity and ease of administration. Although it has been shown that thiamine‐deficient mothers effectively absorb oral thiamine with sharp increases in breastmilk thiamine concentration, their breastfed infants remained thiamine‐deficient after five daily doses of 100 mg oral thiamine supplementation to the mother.^51^ Therefore, symptomatic infants should receive thiamine directly. Intramuscular route can be opted in community settings where immediate intravenous access in infants is problematic. Switching to the oral route should be considered as soon as possible after the initial thiamine bolus.

**Table 3:** Thiamine treatment regimens and response to treatment among infants, children, and adults with varying clinical manifestations of thiamine deficiency disorders in case–control studies and hospital‐based prospective cohorts and retrospective reviews

Study	*n*	Age and country of patient population	Clinical presentations	Thiamine treatment dose and duration	Response to treatment
**Infants and children**					
Rao *et al*.^55^	166	Mean age = 7 months India	Severe respiratory irregularities, ophthalmoplegia, seizures, hypotonia, fever, and vomiting. Associated features: aphonia, choreo‐athetoid movements, arreflexia, and loss of milestones, with head lag	200–300 mg daily, followed by 75 mg daily for 3 months after discharge	Consciousness, respiratory abnormalities, and ptosis improved within 24 hours Head control, tone, involuntary movements, and milestones recovered partially over a few weeks Developmental delay and hypotonia remained at 3–6 months follow‐up in two cases with persistent computed tomography head lesions
Rao and Chandak^27^	55	Mean age 3.9 months India	Tachypnea, chest indrawing, tachycardia, high output heart failure, and pulmonary hypertension, but also hepatomegaly, cough, fever, aphonia, nystagmus, and altered consciousness	75 mg IM twice daily for 5 days	Reversal of right arterial and ventricular dysfunction at 2–3 weeks follow‐up in 19 cases
Coats *et al*.^43^	27	≤7 months Cambodia	Hepatomegaly, respiratory rate ≥40, heart rate ≥140, absence of fever, and at least two of the following: aphonia or dysphonia, wheezing, decreased urine output, recent vomiting, and irritability	100 mg IM for 3 days	Respiratory rate decreased by ≥10 breaths/min in 26% by 24 h and in 38% by 72 hours Heart rate decreased by ≥20 beats/min in 30% by 24 h and in 33% by 72 hours Twenty percent had decreased liver size by ≥1 cm by 72 hours
Porter *et al*.^9^	20	2–47 weeks Cambodia	Hepatomegaly, respiratory rate ≥40, heart rate ≥140, temperature <37.5 °C	100 mg IM for 3 days	Significant decreases in respiratory and heart rate and liver size by 48 hours Two cases with right ventricular enlargement improved within 48 hours
Qureshi *et al*.^53^	23	32 days–4 months India	Tachycardia, irritability in the form of excessive crying and restlessness, moaning, reduced feeding for 1 day and seizures Blood lactate levels were >15 mmol/L in all patients	100 mg IV on admission, and 50 mg IV daily until discharge from hospital	Moaning subsided within 2 h, vacant stare and tachycardia within 4 h, and normalization of breastfeeding within 12 hours Blood lactate <4 mmol/L was attained within 4 hours Hyperechoic putamen reversed at 1‐month follow‐up in eight cases
Wani *et al*.^22^	58	35 days–9 months India	Infantile encephalitic beriberi: altered consciousness, seizures, altered personality, or cognition	100 mg IV on admission to hospital	Regression of basal ganglia hyperechogenicity with almost normal appearance at 2–4 weeks follow‐up in 18 infants and at 4–8 weeks in an additional 8 infants Ten infants with persistent basal ganglia hyperechogenicity showed delayed developmental milestones
Bhat *et al*.^58^	50	1–6 months India	Infants with acute onset encephalopathy: irritability, blephroptosis, gastroesophageal reflux, seizures, right heart failure, vacant stare, and aphonia	100 mg IV daily and 10 mg/day orally after discharge	Improvement in symptoms in median time of 7 hours Eight patients were discharged with some neurological deficits in the form of aphonia, multiple cranial neuropathies, or motor deficits
Sastry *et al*.^56^	231	Mean age 3.2 months India	Fast breathing, chest retractions, irritability, poor feeding, vomiting, aphonia, tachypnea, tachycardia, and hepatomegaly	100 mg IV daily for 3 days	Pulmonary hypertension resolved in 92% of cases within 24–48 hours. Within 6 h, feeding improved and vomiting ceased Tachypnea, tachycardia, and hepatomegaly reduced within 24 hours Aphonia resolved over 3–4 days.
Thankaraj *et al*.^54^	28	Mean age 69 days India	Tachycardia, prolonged capillary refill time, severe respiratory distress, seizures, vomiting, breathlessness, and poor feeding	100 mg IV daily for a minimum of 7 days	Resolution of shock within 24 h and initiation of breastfeeding within 2 days Fourteen infants requiring invasive ventilation could be weaned within 60 h, with 12 infants being extubated within 24 hours Improvement in capillary blood gas measurements within 4–8 hours
**Adults**					
Shah *et al*.^66^	50	23–80 years India	Nonalcoholic Wernicke's encephalopathy mainly presenting with nausea/vomiting, nystagmus, lower limb weakness, ataxia, altered mental status, and memory impairment	300–600 mg IV twice daily for 5–10 days, followed by 100–300 mg/day oral maintenance	Nine patients had residual symptoms after 9 days of treatment, mainly lower limb weakness, ataxia, memory impairment, and psychosis One patient developed Korsakoff psychosis
Koshy *et al*.^70^	24	15–40 years India	Peripartum women with peripheral neuropathy and/or cardiopathy	200 mg IV or IM per day for an average of 7 days, followed by B‐complex (33 mg thiamine) twice daily at discharge	Ninety percent of patients reported improvements in neurological deficits or in nerve conduction studies after an average of 10 days One patient with repeat echocardiogram 1 week after treatment showed improved cardiac output and disappearance of a functional mitral regurgitation
Nilles *et al*.^38^	69	Median age 28 years (range 0–62) Kiribati	Eighty‐three percent of cases were male. Main features were weakness, paresthesia, numbness, pain, or edema of the extremities	100 mg IM daily for 1–3 days, followed by 100 mg oral daily for 3–6 weeks	Ninety‐four percent of cases reported complete or near‐complete resolution of symptoms within 7 days Of cases unable to complete squat tests or heel walk tests, 55–77% could successfully complete within 3–7 days of treatment
Hilal Ahmad *et al*.^69^	29	Mean age 30.2 years India	Peripartum women with peripheral neuropathy	200–500 IV three times daily for 3–5 days, followed by oral thiamine	Within 24–72 h, 27 patients showed improvements in weakness, mental status, ophthalmoparesis, and nystagmus and resolution of edema

IM, intramuscular; IV, intravenous.

The response to thiamine is rapid in infants presenting with cardiovascular manifestations of TDDs, with resolution of shock, irritability, tachycardia, normalization of breastfeeding, and apparent recovery within hours of thiamine administration,^37^, ^52^, ^53^, ^54^ and significant decreases in respiratory rate and liver size within 48 hours.^9^, ^43^, ^55^, ^56^ Reversal of echocardiographic abnormalities has been observed at 2–3 weeks following 75 mg intramuscular thiamine twice daily for 5 days in exclusively breastfed infants presenting to the hospital with cardiac beriberi.^27^ Among 20 Cambodian infants with biochemically confirmed thiamine deficiency (median ThDP = 47.6 nmol/L), two cases with the lowest baseline ThDP concentrations of 24 and 21 nmol/L were found to have mild right ventricular enlargement and severe right ventricular enlargement with severely diminished right ventricular function, respectively, on echocardiographic examination.^9^ All cases were treated with 100 mg intramuscular thiamine per day for 3 days. A repeat echocardiogram at 48 h revealed that the right ventricular enlargement had returned to normal in both cases. Infants and children treated for beriberi or suspected TDDs in the clinical setting are often provided with oral thiamine supplements following recovery and their mothers with 100 mg oral thiamine twice daily for 30 days (Table 2).^57^

A differential response of signs and symptoms has been observed in neurological forms of TDDs in infants and children, and the implications of different dosing regimens on long‐term outcomes are not clear. Bhat *et al*. reported on the treatment outcomes of 50 exclusively breastfed infants with a mean age of 3.2 months presenting with Wernicke's encephalopathy in Kashmir.^58^ Following 100 mg/day intravenous infusion, there was a rapid improvement in symptoms, including blepharoptosis and irritability, although patients with associated aphonia were slow to respond. Neurologic abnormality at discharge, after a median of 6 days in hospital, in the form of aphonia and motor deficit, was observed in 16% of infants. In a retrospective review of cranial ultrasonography of infants (mean age = 3.5 months) admitted to hospital with encephalopathy, 58 infants were found to have thiamine‐responsive encephalopathy, 41 of which had abnormal findings on the cranial ultrasound scan.^22^ Symptomatic response to thiamine was rapid, with tachycardia subsiding in 4 h, and reversal of ophthalmic signs and normalization of breastfeeding within 6 hours. In infants with developmental delay, milestones and tone abnormalities were slow to resolve over a period of 4–14 weeks. Initial basal ganglia lesions recognized on a cranial ultrasound scan at presentation (*n* = 41) were demonstrated to decrease slowly, reverting to a normal appearance within 4–8 weeks in 63% of infants (*n* = 26), 22 of which achieved age‐appropriate milestones, while 4 demonstrated motor delays at follow‐up. In 37% of infants, the basal ganglia showed persistent hyperechogenecity, and 15% showed some atrophy of the basal ganglia with ventriculomegaly at follow‐up. All infants were treated with a 100‐mg intravenous thiamine infusion at admission and daily thiamine for 6 weeks after discharge. Conversely, a daily dose of 100 mg thiamine was insufficient to treat a 13‐year‐old adolescent with Wernicke's encephalopathy derived from prolonged malnutrition during treatment for a neuroblastoma, and only after high‐dose thiamine replacement therapy of 500 mg three times daily (1500 mg/day) did symptoms resolve and the brain MRI appeared normal after 2 months of therapy.^59^ These observations suggest that, compared with the cardiac form of TDDs, neurological manifestations may require higher doses of thiamine replacement therapy and a longer recovery time.

Children with SAM are likely at risk of thiamine deficiency, and this can be further induced during refeeding by increased thiamine demand due to glucose utilization.^48^ The average daily intake of thiamine is only 1–2 mg if ready‐to‐use therapeutic food (RUTF) or therapeutic milk (F‐75 or F‐100) is given in the stabilization phase as per World Health Organization protocols, which is just one to four times the recommended daily intake. Infants under 6 months of age with SAM do not receive RUTF, F‐75, or F‐100 but only receive breastmilk or specifically diluted F‐100 or F‐75 when regular infant formula is not available to supplement breastfeeding. In view of the high thiamine needs, there have been recent calls for the reformulation of therapeutic foods with increased thiamine content^60^ or to treat critically ill SAM patients with pharmacological doses of thiamine (intravenous or oral), according to clinical indications in the early stages of refeeding.^61^ For example, recent guidelines from the American Society for Parenteral and Enteral Nutrition recommend 2 mg/kg thiamine, to a maximum of 100–200 mg/day, before feeding or dextrose‐containing fluids are initiated, followed by thiamine supplementation for 5–7 days or longer in high‐risk patients.^62^

#### Adults

Experimental and clinical data indicate that, compared with intravenous doses, orally administered thiamine is less effective at increasing blood thiamine and may not be suitable for treating neurological conditions associated with TDDs.^63^, ^64^ Successful resolution of Wernicke's encephalopathy and Wernicke–Korsakoff syndrome has been described using a wide range of empirically derived intravenous dosages. In a double‐blind randomized controlled triad among alcohol‐dependent patients without the clinical triad of severe Wernicke–Korsakoff syndrome (*n* = 107), Ambrose *et al*. assessed the efficacy of daily doses of 5, 20, 50, 100, and 200 mg intramuscular thiamine hydrochloride for 2 days, with assessment of effect on the third day by a neuropsychological test.^65^ The authors did not report the effect of treatment on ocular palsies and ataxia, but concluded that the patients receiving the 200 mg dose demonstrated superior performance on the delayed alternation test, an assessment of working memory, although there was no apparent dose–response effect of treatment. However, there is little information about the reliability of this test in humans, and the results should be interpreted with caution due to the small sample size randomized to each treatment arm, the high dropout rate, and the short duration of treatment. In a series of 50 adult cases of nonalcoholic Wernicke's encephalopathy in northern India, there was partial or complete improvement in symptoms in 49 patients following treatment at a dose range of 300–600 mg intravenous thiamine twice daily for 5–10 days, followed by oral maintenance of 100–300 mg/day.^66^ One patient did not show any response and went on to develop Korsakoff psychosis. After 9 days of treatment, nine patients had residual symptoms, the most common being lower limb weakness, ataxia, memory impairment, and psychosis. It is recommended that further continuation of treatment should be titrated by clinical state and should be continued until there is no further improvement in signs and symptoms.^67^ A recent re‐emergence of TDDs in the Pacific Islands of Kiribati, affecting mainly adult males, showed that within 7 days of starting treatment (100 mg intramuscular for 1–3 days followed by 100 mg oral daily doses for 3–6 weeks), 94% of cases reported complete or near resolution of symptoms, with the majority able to subsequently perform squat tests and heel walk tests.^38^

Gestational Wernicke's encephalopathy caused by hyperemesis gravidarum–induced thiamine deficiency has been reported to respond to high‐dose thiamine treatment. A case report of a 25‐year‐old pregnant woman in India demonstrated dramatic improvement in mental state and nystagmus within 48 h of initiation of treatment (900 mg every 24 h in divided doses for 5 days).^68^ After 6 weeks, the visual signs, gait, and reflexes regained their normal status. A repeat MRI revealed complete resolution of the lesions in comparison with the pretreatment image.

In a retrospective hospital‐based study in Kashmir, of 27 peripartum women with a history of hyperemesis gravidarum or nausea with poor intake and thiamine deficiency–related peripheral neuropathy, 19 patients improved with intravenous thiamine doses of 200–500 mg three times daily for 3–5 days followed by oral thiamine.^69^ A positive response to thiamine was assessed by subjective and objective improvement of weakness and amelioration of edema. Improvements in symptoms were noted within 24–72 h of thiamine administration. Similarly, 24 peripartum women presenting to a hospital in rural Assam with features consistent with thiamine deficiency–related polyneuropathy and/or cardiopathy were treated with 200 mg intravenous or intramuscular thiamine for an average of 7 days, followed by a B‐complex oral supplement (containing 33 mg thiamine) twice daily until follow‐up.^70^ Ninety percent of patients reported improvements in neurological deficits or improvements in nerve conduction studies after an average of 10 days. Of six patients with abnormal echocardiography at admission, only one patient had a repeat echocardiogram 1 week after parenteral treatment, which showed improvements in cardiac output and disappearance of a functional mitral regurgitation.

The optimal treatment regimen for cardiac beriberi in adults has not been well studied and varies depending on the severity of presentation. Helali *et al*. reported two cases of heart failure due to cardiac beriberi with severely reduced left ventricular ejection fraction, one of whom improved after 100 mg/day oral thiamine supplementation and the second with daily 100 mg intravenous supplementation.^71^ Both cases had normalization of ejection fraction on subsequent echocardiogram. Therefore, treatment with 100 mg oral or intravenous thiamine has been shown to successfully treat cardiac beriberi in adults. The mode of administration may be guided by severity of symptoms and consequently the requirement of admission to a clinical setting for intravenous treatment.

#### Safety profile

Thiamine is well tolerated even in high doses (≥500 mg/day). The dilution should preferably be done in saline. Intravenous dextrose solutions may increase thiamine requirements and may worsen symptoms in patients who are thiamine deficient. The use of normal saline to dilute thiamine decreases the likelihood of a rare anaphylactic reaction.^72^ A prospective evaluation of thiamine hydrochloride given as a 100 mg intravenous bolus dose in 989 patients (1070 doses) reported 12 adverse reactions (1.15%), of which 11 were deemed minor reactions consisting of transient local irritation, and one major reaction consisting of generalized pruritus.^73^ Thiamine should preferably be administered intravenously and the intramuscular route for thiamine repletion should be limited to patients without intravenous access in emergency situations, based on the pain and discomfort of multiple administrations.^67^

#### Consideration of concomitant treatments

Magnesium is an essential cofactor in the conversion of thiamine to its active form ThDP. Both ThDP and magnesium are required as cofactors for pyruvate dehydrogenase, a key enzyme in the Krebs cycle. Thus, hypomagnesemia can have a dual negative effect on thiamine metabolism, and low status of thiamine and/or magnesium may compromise enzyme activity, resulting in altered metabolism of glucose and increased lactate production.^48^, ^74^ Without correction of concomitant hypomagnesemia, which can be common among critically ill patients, there may be impaired utilization of thiamine. Case reports in hypomagnesemic adult patients with Wernicke's encephalopathy and Crohn's disease demonstrated that despite therapeutic doses of thiamine, symptoms of thiamine deficiency could not be suppressed until the low magnesium status was corrected.^75^, ^76^, ^77^ Chronic alcoholic patients who received magnesium alongside thiamine had greater increases in the thiamine biomarker ETK activity than those who received thiamine alone, suggesting that coadministration of magnesium and thiamine may be required for enabling full efficacy of thiamine treatment.^78^ Hence, intramuscular coadministration of magnesium (1–2 mL of a 50% solution) can be given to patients who are at risk of hypomagnesemia or in those with thiamine refractoriness.^13^, ^75^

### Prevention at the community level

As infants are particularly vulnerable to TDDs in the first year of life, and exclusively breastfed infants of thiamine‐deficient mothers are at the highest risk,^79^ routine preventive supplementation to at‐risk pregnant and lactating women may be warranted. Breastmilk thiamine concentrations and subsequent infant status are dependent on maternal thiamine intake and status, and maternal thiamine deficiency rapidly results in low breastmilk thiamine concentrations.^40^ This has been demonstrated among lactating women in LMICs,^51^, ^80^ but also in higher‐income countries when maternal thiamine intake during pregnancy was low.^81^ Thiamine provided through fortified foods and supplements has been shown to rapidly increase breastmilk thiamine concentrations and maternal status in thiamine‐deficient populations.^51^, ^82^, ^83^, ^84^ Luxemburger *et al*. demonstrated the benefits of supplementing Karen pregnant women in the Maela refugee camps where a large peak in infant mortality at 3 months of age had been recognized, and infantile beriberi accounted for 40% of all infant deaths.^52^ Supplementation of symptomatic pregnant women with 100 mg daily until delivery and 10 mg weekly until 9 months postpartum for all lactating women was associated with a reduction in the infant mortality rate from 183 to 78 per 1000 live births and in mortality attributed to beriberi from 73 to 5 per 1000 live births. Additionally, the case fatality of infantile beriberi declined from almost 100% to 7%. It may, therefore, be important to supplement mothers from the last trimester of pregnancy and throughout the period of breastfeeding, although the optimal timing, dose, and duration of supplementation require further investigation. Despite five daily doses of 100 mg thiamine significantly increasing maternal whole blood ThDP and breastmilk thiamine concentrations among Cambodian women, 94% of their infants remained thiamine deficient according to a cutoff of 70 nmol/L.^51^ Although safe, an oral dose of 100 mg is significantly greater than the recommended intake of 1.5 mg per day for lactating women,^85^ and optimal dosing regimens of lactating mothers remain unclear. Intestinal thiamine absorption occurs via two mechanisms: an active, carrier‐mediated saturable process at low concentrations (<1 μmol/L)^86^ and an unsaturable passive diffusion process at higher concentrations.^87^ In human studies, single oral doses of thiamine greater than 2.5–5 mg have been shown to go largely unabsorbed,^64^, ^88^ although more recently, high blood thiamine concentrations were achieved in healthy adults with doses of oral thiamine hydrochloride up to 1500 milligrams.^87^ Furthermore, it has been estimated that only 0.05–0.35% of thiamine in a single dose oral supplement is secreted into breastmilk and thus available to the breastfed infant.^89^ As such, high‐dose preventive supplementation of lactating mothers may not be necessary and it may be more appropriate to supplement the mother at doses closer to the recommended intake and for a longer duration to ensure sufficient thiamine is received by the breastfed infant, although this may depend on locally available supplements. Other factors, such as overall nutritional status, dietary intakes, and consumption of antithiamine compounds, may affect thiamine absorption. A high prevalence of biochemical thiamine deficiency was found in Karen refugee women at 3 months postpartum, despite the provision of 100 mg thiamine supplements during pregnancy if symptomatic of thiamine deficiency (principally peripheral paresthesia) and 10 mg thiamine per week during lactation to all women, alongside food rations of thiamine‐rich foods.^35^ Despite the biochemical thiamine deficiency in these mothers, breastmilk thiamine concentrations within the normal range were reported in human milk, and a lower prevalence of thiamine deficiency in cord blood compared with maternal blood at delivery indicates preferential delivery of thiamine to the breastmilk and sequestration of thiamine by the fetus, as suggested by others.^81^, ^90^ Maternal deficiency may have been due to the widespread consumption of fermented fish, betel nuts, and fermented tea leaves, all known to contain antithiamine compounds that inhibit the absorption and bioavailability of thiamine and exacerbate thiamine deficiency even in the presence of adequate intakes.^91^ In such contexts, where maternal dietary thiamine intakes are insufficient due to low dietary diversity, where consumption of thiamine antagonists or thiaminases is widespread, or where traditional, highly restrictive postpartum diets are commonplace,^92^, ^93^ pregnant and lactating women may require concurrent nutrition counseling and behavior change communication in order to optimize thiamine supplementation programs.

Although no association between low thiamine status and the presence of clinical signs of deficiency (e.g., peripheral paresthesia) has been reported among pregnant women^35^ or breastfeeding mothers,^27^ widespread thiamine supplementation through antenatal care programs should be considered in high‐risk areas in view of the high risk of mortality among breastfed infants. Attention should also be given to the optimal method of administration, either through a thiamine or B‐complex supplement as an addition to the current antenatal iron folic acid supplementation program, or the introduction of a multiple micronutrient supplement. Laos and Myanmar, both countries with low dietary thiamine intakes and reports of infantile beriberi, distribute thiamine supplements during pregnancy and lactation, either as 100 mg thiamine supplements (in addition to iron folic acid in Laos)^94^ or as multiple micronutrient supplements containing 1.4 mg thiamine (the recommended intake for pregnant women) in Myanmar.^10^ However, these programs currently only cover the first 3 months postpartum and do not cover the whole period of recommended exclusive or continued breastfeeding. Ideally, supplementation should continue for 6 months or beyond, until there is assurance that the child is receiving enough thiamine from complementary foods. Regardless of the method of maternal supplement administration chosen, it is crucial for ongoing monitoring and evaluation programs to appraise both the coverage and adherence of such efforts.

Pregnant and lactating women are key groups to target in order to reduce the risk of TDDs; however, depending on the context, preventive supplementation may offer benefits to other at‐risk population subgroups, although few supplementation programs exist. While supplementation can be effective at the individual level or targeted to specific population subgroups (e.g., pregnant women through antenatal care programs), it can be less effective at the population level, particularly in LMICs, due to low uptake and poor adherence to long‐term supplementation regimens, coverage, supply chain and healthcare infrastructure issues, and cultural acceptability. Thus, supplementation programs may be a short‐term solution while longer‐term food‐based programs are implemented. Large‐scale mandatory food fortification may be a preferred and more sustainable method to improve the thiamine status of a population at high risk of deficiency, with limited behavior change needed. Thiamine fortification strategies in low‐ and middle‐income settings are reviewed in detail elsewhere in this special issue.^95^

### Current global scenario of thiamine deficiency

TDDs are beginning to be recognized as an important public health issue in several LMICs, especially in South and Southeast Asia in the last decade. Since early 2000, several case reports and case series on TDDs have thrown light on the fact that TDDs are still rampant in at‐risk populations in several countries. A few countries, such as India, have documented different clinical presentations of TDDs in the same general population (Table 1). Other countries, such as Thailand, have documented both dry and wet beriberi in specific populations, including fishing crews,^96^ factory laborers,^97^ and prisoners.^98^

The prevalence and global burden of thiamine deficiency is poorly documented, since, to date, only Cambodia has included the assessment of thiamine status in their recent nationally representative nutrition survey,^99^ and the 2017–2018 Myanmar Micronutrient and Food Consumption Survey included an assessment of thiamine status among women of reproductive age and pregnant and lactating women. Few countries, such as Thailand, have the facilities to routinely perform standard biomarker assessments, and biomarker assessment has otherwise predominantly been used in research settings in most countries, now including Bhutan. Johnson *et al*. recently reviewed the prevalence of thiamine deficiency in LMICs, suggesting that thiamine deficiency is endemic in Southeast Asia, but also noting that several reports of thiamine deficiency have emerged from parts of South Asia, Africa (see also Ref. 100), and Latin America, raising concerns that thiamine deficiency may be an important public health concern in these regions.^12^

Given the wide variability in clinical presentation, large pool of asymptomatic groups, and the lack of point‐of‐care diagnostic tests and clear thiamine biomarker cutoffs, countries where TDDs appear to be endemic may utilize surrogate markers to identify at‐risk populations. Normally, in a population, there is a steep decrease in infant mortality throughout the first 6 months. A typical feature of infantile beriberi is that instead of infant mortality decreasing after the first month, it remains high or even peaks at about the third month, with a second peak around the sixth month.^79^, ^99^ Hence, determining age‐specific infant mortality rates in countries with high rates of infant mortality can be a useful tool to screen at‐risk populations.

It is to be noted that, although clinical cases largely focus on infantile beriberi and its contribution to infant mortality rates in LMICs, cardiac beriberi among pregnant and lactating women has been poorly studied and may possibly be a preventable cause of maternal mortality in these countries. Studies have identified pregnant and lactating women to be at risk of developing clinically overt thiamine deficiency due to increased requirements of thiamine during this period.^38^, ^70^, ^101^ A health facility in rural Assam that manages infantile beriberi and neurologic TDDs among peripartum women has also documented thiamine‐responsive cardiomyopathy among peripartum patients and now includes parenteral thiamine in addition to routine heart failure therapy, which is similar to the management of infantile beriberi, may have been lifesaving (R. Koshy, personal communication).

The extent of research in countries on TDDs has largely relied on public health interest and extent of involvement of the government at the district or national level. Countries with documented TDDs have started to take initial steps to introduce national surveillance and thiamine deficiency prevention programs, although the feasibility and efficacy of such programs has yet to be evaluated, in part due to the complexities in the identification and diagnosis of clinical TDDs and biochemical thiamine deficiency. For example, a 2014 nationally representative survey reported beriberi to be the second leading cause of death among children under 5 years of age in Myanmar, accounting for 17% of deaths among infants aged 1–12 months.^102^ In response, Myanmar has since established a thiamine supplementation program for pregnant and lactating women and has developed a rice fortification national policy that is currently being scaled up.

### The way forward

There has been significant progress in the scientific understanding of thiamine deficiency, yet many infants, children, and adults still suffer from TDDs, especially in resource‐limited areas of Asia. Without emphasis on active research to study TDDs in at‐risk populations, TDDs will continue to be a neglected public health disease and a preventable cause of morbidity and mortality in LMICs this century. Based on this review of clinical TDDs, several future foci are appropriate as we move toward reducing morbidity and mortality caused by thiamine deficiency.

First, we must develop and complete further studies of the pathophysiology of thiamine deficiency. There are not yet good studies looking at the genetics of patients suffering from TDDs, and it could be that genetic variations increase the risk of adverse outcomes of inadequate thiamine nutrition. If so, interventions could be directed toward at‐risk population groups. In addition, it is not clear when and how CNS pathology develops in some, but not all, thiamine‐deficient children. Studies of metabolic mediators would help understand the process, and natural history studies would help better understand the clinical course of basal ganglia changes with thiamine deficiency, again pointing toward more effective identification and management of affected individuals. Also, it is not clear how thiamine interacts with other factors (nutritional and environmental) to determine which affected patients develop cardiac versus respiratory versus neurological complications of thiamine deficiency. Clinical studies could help identify better timing of necessary treatments.

Second, the clinical diagnosis of TDDs remains extremely challenging. Ongoing studies are in progress,^46^ and the results will be necessary to help better identify patients in desperate need of thiamine treatment. Diagnostic measures, ideally, would not depend on high‐technology testing but would be based on identifiable clinical features. We must complete studies evaluating the clinical diagnosis of thiamine deficiency states and then educate healthcare professionals about those findings.

Third, there is incomplete knowledge of the most effective dosing and treatment of thiamine for affected patients. We must engage in further studies that compare oral versus parenteral treatment at various doses with differing durations of treatment. While thiamine is nearly without danger, consistent evidence‐based treatment guidelines must be developed.

Fourth, population‐level thiamine assessments are needed to guide either supplementation or food fortification programs to at‐risk populations and subgroups to reduce the number of people who ever develop clinical manifestations of thiamine deficiency.

Finally, political engagement is necessary and should be encouraged. Many regions seem to have high levels of thiamine deficiency, while lacking documentation of the extent of the problem. Thiamine deficiency, like other “silent” micronutrient deficiencies, can easily go undetected if epidemiological and monitoring programs are not implemented. Professional education is needed to raise the awareness of TDDs in areas where these conditions are currently going unrecognized, such as in parts of Africa.^100^ Then, policy development will be required to make treatments and treatment regimens available and accessible in areas of need.

With concerted effort and multinational collaboration, it is foreseeable that the current unconscionable levels of morbidity and mortality due to thiamine deficiency can be reduced. The way forward is clear, and the time is ripe for ongoing research, monitoring, care, and policy implementation.

## Author contributions

All authors contributed to and approved the final version of the manuscript.

## Competing interests

The authors declare no competing interests.
